# A Chinese Family With Cerebral Cavernous Malformation Caused by a Frameshift Mutation of the *CCM1* Gene: A Case Report and Review of the Literature

**DOI:** 10.3389/fneur.2022.795514

**Published:** 2022-04-04

**Authors:** Wenyu Liu, Ming Liu, Di Lu, Jiwei Wang, Zexin Cao, Xuchen Liu, Zichao Feng, Bin Huang, Xinyu Wang

**Affiliations:** ^1^Department of Neurosurgery, Qilu Hospital, Cheeloo College of Medicine, Shandong University and Institute of Brain and Brain-Inspired Science, Shandong University, Jinan, China; ^2^Key Laboratory of Brain Function Remodeling, Qilu Hospital of Shandong University, Jinan, China

**Keywords:** familial cerebral cavernous hemangioma, *KRIT1/CCM1* gene, case report, literature review, Chinese family

## Abstract

**Background:**

Familial cerebral cavernous malformation (FCCM) is a vascular malformation disease closely linked to three identified genes: *KRIT1/CCM1, MGC4607/CCM2* and *PDCD10/CCM3*. Over the past decade, a few cases of cerebral cavernous malformation (CCM) caused by different gene mutations have been reported in Chinese families. Herein, we introduce a Chinese family affected by FCCM due to a kind of *KRIT1/CCM1* frameshift mutation. At the same time, a literature review was conducted to identify case reports of familial cerebral cavernous malformation.

**Case presentation:**

The proband in the family in question demonstrated a series of clinical symptoms and features, including headache and bleeding. The proband was hospitalized for headache twice and, both times was examined under suspicion of CCM and received surgical treatment. Magnetic resonance imaging results showed that the proband had multiple intracranial vascular lesions, including on the brain, brainstem, and cerebellum. Genetic test results showed that the classic *KRIT1* gene in the proband had a pathogenic mutation. The family members of the proband also showed typical cerebral cavernous malformation when considering clinical manifestations, magnetic resonance imaging findings and genetic test results.

**Conclusions:**

We report a case of Chinese FCCM and its associated symptoms with *CCM1*-deletion mutations in China. Our findings deepen our understanding of CCM mutations and related phenotypes, the investigation results of this clinical experiment further show that the gene mutation form we reported plays an important role in human FCCM, and this trial investigation is beneficial for genetic counseling for CCM patients.

## Introduction

Cerebral cavernous malformations (CCMs) are relatively rare vascular anomalies occurring in single or multiple sites (1) in the brain and spinal cord where small blood vessels are enlarged with irregular structures (2). A large autopsy study showed that the prevalence of CCM accounted for 0.1 to 0.5% of the total population (3), while other research has indicated that CCM influences ~10 to 20% of all cerebral vascular abnormalities (4). The disease can occur sporadically or by way of familial autosomal dominant inheritance without complete penetrance (5), with most cases having the former nature. The age of onset of CCM is typically 20 to 30 years of age or older. About 40% of CCM patients can be clinically asymptomatic, while others may suffer from repeated headaches, hemorrhagic strokes, seizures, or focal neurological deficits (6). Currently, magnetic resonance imaging (MRI) is commonly used to diagnose CCM (7), and the characteristic appearance of this phenomenon on MRI appears with concentrated mixed signal strengths and a hemosiderin-containing ring, and performs best on the inversion recovery sequence of T2-weighting and fluid attenuation. It is a complex undertaking to surgically remove multiple lesions at once, making the successful treatment of FCCM difficult (8).

Genetic locus heterogeneity has also been demonstrated by linkage mapping to additional loci on chromosomes 7p13–15 (*CCM2*, OMIM 603284) and 3q25.2–27 *(CCM3*, OMIM 603285) (9). To our knowledge, *KRIT1/CCM1* encodes for the krev interaction trapped 1 (Krit1) protein. Mutations that occur mainly in *CCM1* can cause the stop codon to terminate prematurely, resulting in truncation of the protein (10).

Reports of FCCM in the Chinese population are limited in number, and most FCCM families that have been reported are Mexican/Hispanic (11, 12). In this article, we describe a frame-shift *CCM1* mutation in exon 14 leading to a premature stop codon in a Chinese family with incomplete penetrance of the disease, which presents further evidence for phenotypic heterogeneity.

## Case Series

The proband was a 52-year-old male, diagnosed with intracranial cavernous hemangioma at the age of 30 years because of an intracranial hemorrhage. Four years ago, he had experienced an intracranial hemorrhage again in another cavernous hemangioma, and the lesions were removed by craniotomy on both occasions. He mentioned that several of his sisters had similar manifestations as he did. Considering the proband's family as one with FCCM, we therefore analyzed and herein report on the following familial case series.

## Patients and Methods

### Patients

**Figure 1:**
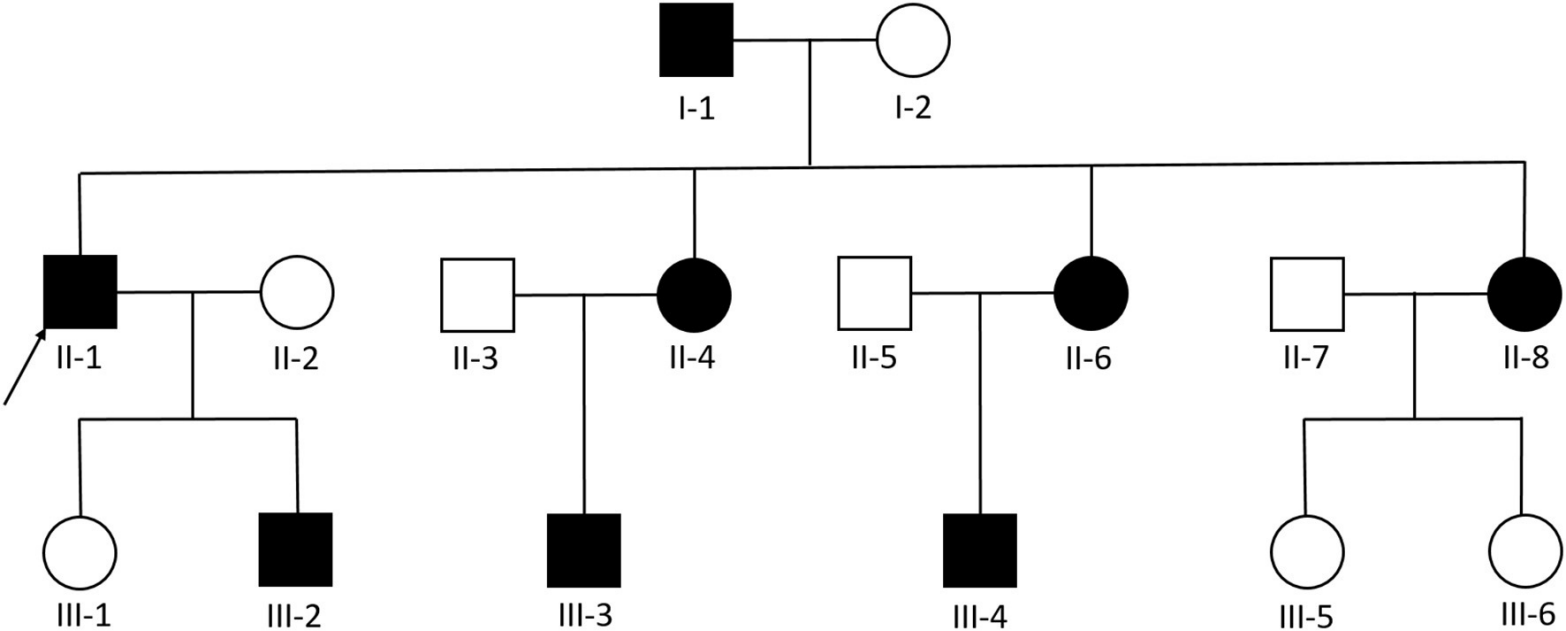
Pedigree diagram of the Chinese family with familial cerebral cavernous malformation, a heterozygous frameshift mutation described as c.1362_1363del (p.Gln455fs) was found in exon 14 of *CCM1*. The arrows designate the index patient (proband); filled squire = affected; unfilled square = unaffected.

We examined 11 members of a family of Chinese descent (Figure 1), with the help of the proband's son, and we established the familial pedigree. All participants signed written informed consent forms before blood sampling and genetic analysis, then we collected peripheral blood samples from members of the family. We checked the presence and location of central nervous system lesions by MRI. The study was approved by the Ethics Committee of Qilu Hospital of Shandong University.

### Methods

Genomic DNA was extracted from the peripheral blood cells (II-1) of the proband, and we used polymerase chain reaction to amplify all coding exons of the three CCM genes (*CCM1, CCM2*, and *CCM3*). We used design primers that encompassed each exon and its respective exon intron-intron splice sites. The amplification primer was used as a sequencing primer, and the sequencing results were subjected to BLAST analysis (http://blast.ncbi.nlm.nih.gov/Blast.cgi). The primer sequences are listed in Table 1. The specific experimental steps were “Sample reception → DNA extraction → library construction (gDNA concentration detection and dilution → end repair and A base addition → adapter ligation and purification of ligation products → library fragment screening → PCR amplification and purification of amplified products → library hybridization and Capture → Product amplification and purification after capture) → On-machine sequencing (On-machine reagent information recording and preparation → Instrument cleaning → On-machine library preparation) → Bioinformatics analysis(details as follows).” First, we obtained fastq data from illumine sequencing machine (NovaSeq6000/HiSeq2500) by using Bcl2fastq (v2.0.1), so the acquired data could be processed by Trimmomatic (Version 0.36) to remove low-quality reads, bases, and trimming adaptors. We then cleaned the data and processed them with the Burrows–Wheeler Aligner (BWA) for alignment on a reference sequence of hg19, using the Genome Analysis Toolkit (GATK) for variant calling. Finally, the Variant Call format (VCF) files were analyzed using the ANNOVAR tool, which contains annotation databases, such as the 1000 genome databases, dbSNP database (dbSNP; http://www.ncbi.nlm.nih.gov/SNP), ClinVar database (ClinVar; http://www.ncbi.nlm.nih.gov/clinvar), the Polymorphism Phenotyping v2 database (Polyphen-2; http://genetics.bwh.harvard.edu/pph2), and the Sorting Intolerant from Tolerant database (SIFT; http://sift.jcvi.org). At the same time, the mutations found in the proband were searched for in the other family members.

**Table 1:** The primer sequence of this single gene sequencing.

Upstream primer sequence	TTCTACCAACCCACTCCCA
Downstream primer sequence	CAACAGATTCTCACTTAAAACAGTG

## Results

### Clinical Investigation

A three-generation Chinese family with nine members affected by familial CCM was identified.

**Figure 2:**
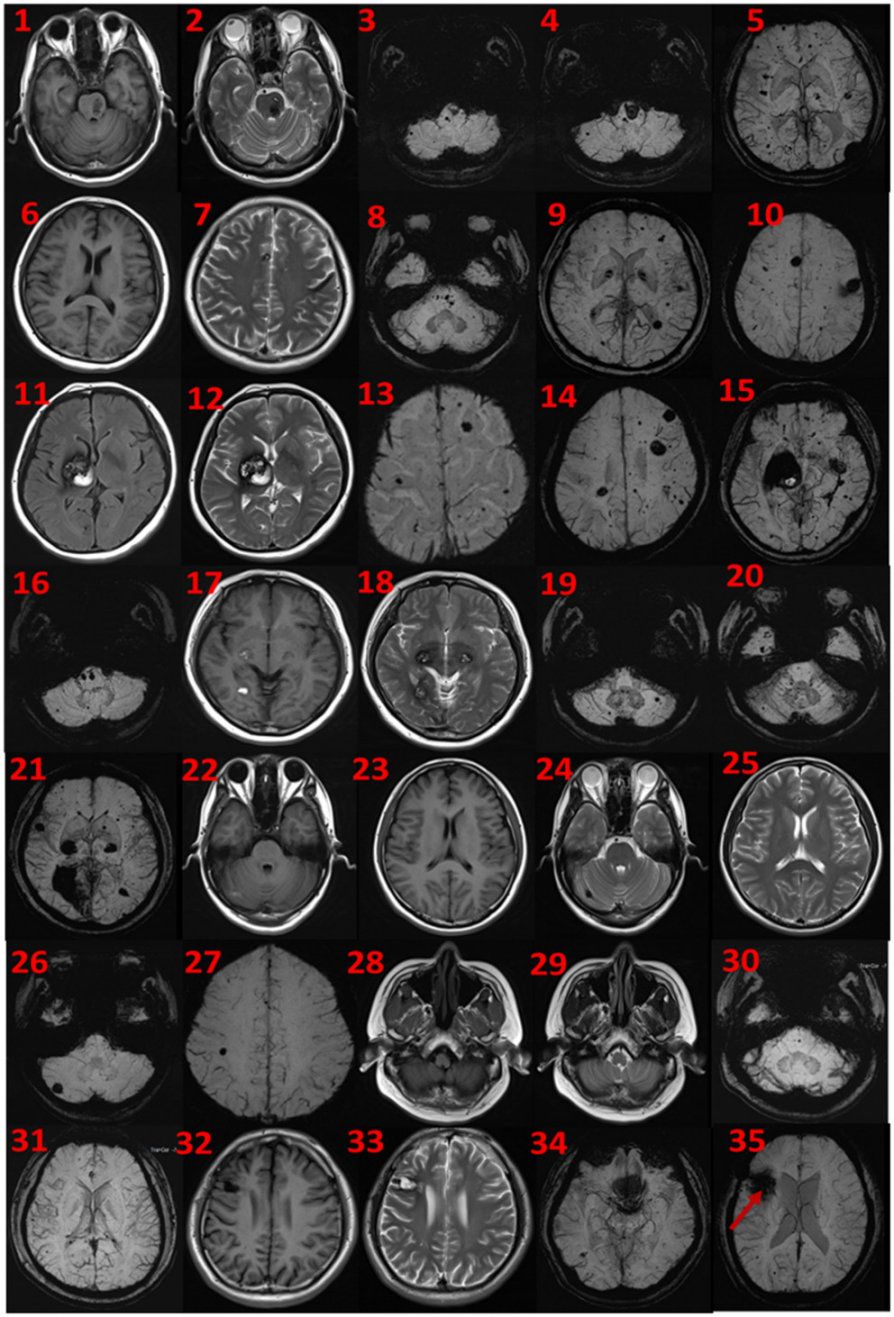
MRI findings. Cerebral MRI in subjects II-1 (1–5), II-4 (6–10), II-6 (11–16), II-8 (17–21), III-2 (22–27), III-3 (28–31), and iii-4 (32–35). 1–5 the MRI imaging showing there are multiple lesions in bilateral cerebellar hemispheres, brainstem area, left thalamus, and white matter areas in both cerebral hemispheres. 6–10 the MRI imaging showing there are multiple lesions in the brain stem and bilateral cerebral hemispheres. 11–16 the MRI imaging showing there are multiple lesions in the cerebellum, brainstem, and bilateral cerebral hemispheres, the large lesions are located in the right basal ganglia area, with a slight space–occupying effect. 17–21 the MRI imaging showing there are multiple lesions in bilateral cerebellar hemispheres, brainstem, bilateral thalamus, and bilateral cerebral hemispheres. 22–27 the MRI imaging showing there are lesions in the right cerebellar hemisphere and the right parietal lobe. 28–31 the MRI imaging showing there are multiple lesions on both sides of the brain, and the arrow points to the postoperative manifestations. 33–35 the MRI imaging showing there are multiple lesions in the brain stem and bilateral cerebral hemispheres.

The proband was a 52-year-old male. His first symptom 30 years ago was a simple headache. He went to the local hospital for imaging examination and found multiple intracranial cavernous hemangioma with a small amount of bleeding (Unfortunately, the specific inspection method and lesion location are unknown). At that time, the cavernous hemangioma at the bleeding site was surgically removed. After the resection, he took Yangjiao granules for 2 to 3 years, and the pain symptoms basically disappeared. However, 4 years ago, he accidentally bumped his head at work and got a headache again without any other symptoms, another imaging examination revealed that a cavernous hemangioma in his skull had slight bleeding. He was resurgically removed the cavernous blood vessels at the bleeding site and recovered well after the operation, and except for numbness on the left face after a short period of time after the operation, he had no other discomfort, finally, the numbness on the left face gradually relieved on its own without any special treatment. The doctor considered that the postoperative symptom was the microbleeding caused by overwork and finally absorbed by itself, or the short-term sequelae caused by the operation, and recommended him to follow-up regularly. Since then, he has not had head symptoms again. The imaging examination of this trial shows multiple cavernous hemangioma in both cerebral hemispheres, as shown in Figure 2 (1–5). The proband's father was 76 years old. He hit his head at work when he was 65 years old and underwent craniocerebral imaging, and multiple cavernous hemangiomas were found in the brain (the specific location and manifestations of the lesions were unclear). So far, he has no obvious symptoms, and according to the doctor's recommendation, he has never given any special treatment to the intracranial cavernous hemangioma. Later, after 5 years of regular follow-up (Simple telephone follow-up), no obvious changes in the lesions were found. Due to his hunchback and some personal reasons, he did not have the latest MRI examination during this study, and the results of the previous cranial imaging examinations were not found. The daughter of the proband is 32 years old and has had no obvious symptoms so far, and her latest MRI examination does not reveal any abnormalities, and the son of the proband son has never had obvious head discomfort, but he once had multiple intracranial cavernous hemangioma in a cranial MRI when he needed a physical examination due to work. Based on his clinical manifestations and imaging data, the doctor recommended no special treatment for hemangioma. He has not usually received MRI of the brain. The results of this imaging examination suggests that he has multiple cavernous hemangioma in the right cerebellar hemisphere and right prefrontal lobe, as shown in the 22–27 diagram of Figure 2. The eldest sister of the proband is 50 years old and, last year, experienced stutter and salivation when the right side of her head was tilted without an obvious cause, she therefore underwent an MRI examination, prompting the diagnosis of CCM. Following her diagnosis, she received conservative treatment at the local hospital and was observed carefully, she gradually recovered, and was discharged from the hospital. Now she is being followed up with regularly. Her latest MRI examination mainly reveals multiple cavernous hemangioma in the brain stem and bilateral cerebral hemispheres, as shown in the 6–10 diagram of Figure 2. The son of the proband's eldest sister has never had obvious head discomfort, nor has he undergone a cranial examination. His latest MRI examination revealed multiple cavernous hemangiomas in the right cerebellar hemisphere and right prefrontal lobe, as shown in the 28–31 diagram of Figure 2. The second sister of the proband is now 47 years old, and 2 years ago, she experienced blurred vision on the right side without any obvious cause, after conservative treatment, her vision improved, and she is currently taking vincamine orally and being followed up with regularly. Her latest MRI results mainly indicate multiple cavernous hemangiomas in the cerebellum, brain stem, and bilateral cerebral hemispheres, as shown in the 11–16 diagram of Figure 2. Her son is 28 years old, and experienced a seizure when he was 13 years old, which was stiff and convulsive, at the time, an imaging examination revealed CCM, then he was treated conservatively and taken carbamazepine orally. The drug was eventually stopped because his symptoms did not relapse, however, 2 years ago, he experienced another epileptic seizure with the same symptoms as the previous one, and an imaging examination revealed multiple cavernous hemangiomas in the brain, one of which was bleeding, so he underwent surgery and the cavernous hemangioma was removed. Since the operation, he has been taking sodium valproate sustained-release tablets regularly to prevent seizures. He experienced epilepsy three months after surgery and once in May 2019, but is now asymptomatic and is followed up with regularly. His latest cranial MRI examination reveals multiple cavernous hemangiomas in the brain stem and bilateral cerebral hemispheres, as shown in the 32–35 diagram of Figure 2. The third sister of the proband is 44 years old, at the age of 27 years, she had no obvious cause for general fatigue, and an imaging examination revealed multiple intracranial cavernous hemangiomas, one of which had ruptured and was bleeding. She underwent surgery to remove the cavernous hemangioma at the lesion, and now she walks with a slight limp, favoring the left half of her body. Her latest MRI examination reveals multiple cavernous hemangiomas in both cerebellar hemispheres, brainstem, bilateral thalamus, and bilateral cerebral hemispheres, as shown in the 17–21 diagram of Figure 2. The genetic analysis of the two daughters of the proband's third sister did not find the sequence of the mutant gene, and the previous MRI results of the brain did not indicate abnormalities, so MRI was not performed in this test. Table 2 shows the incidence, diagnosis, treatment, and prognosis of the family members. Representative MRIs of the family members with imaging changes can be found in Figure 2

**Table 2:** The incidence, diagnosis, treatment, and prognosis of family members.

**Items Patients**	**Age of first onset (years)**	**Form and symptoms of first onset**	**All experienced symptoms**	**Total number of attacks**	**Surgical treatment**	**Current health status**	**MRI results**	**Carrying pathogenic genes**
I-1	--	--	No	0	No	good	Multiple hemangiomas	Yes
II-1	22	Headache after head bump	Headache, intracranial hemorrhage	2	Yes	good	Multiple hemangiomas	Yes
II-4	49	Sudden stuttering, salivation, head tilting to the right	Stuttering, salivation, head tilt, cerebral hemorrhage	1	No	good	Multiple hemangiomas	Yes
II-6	45	Sudden blurred vision in the right eye	Blurred vision in the right eye,cerebral hemorrhage	1	No	good	Multiple hemangiomas	Yes
II-8	27	Sudden general fatigue	General fatigue, cerebral hemorrhage	1	Yes	Limp, skewed to the left	Multiple hemangiomas	Yes
III-1	--	--	No	0	--	--	No obvious abnormalities	Yes
III-2	--	--	No	0	No	Good	Multiple hemangiomas	Yes
III-3	--	--	No	0	No	Good	Multiple hemangiomas	Yes
III-4	13	Sudden seizure	Seizures, cerebral hemorrhage	2	Yes	Occasional seizures	Multiple hemangiomas	Yes
III-5	--	--	--	--	--	--	--	No
III-6	--	--	--	--	--	--	--	No

### Genetic Analysis

**Figure 3:**
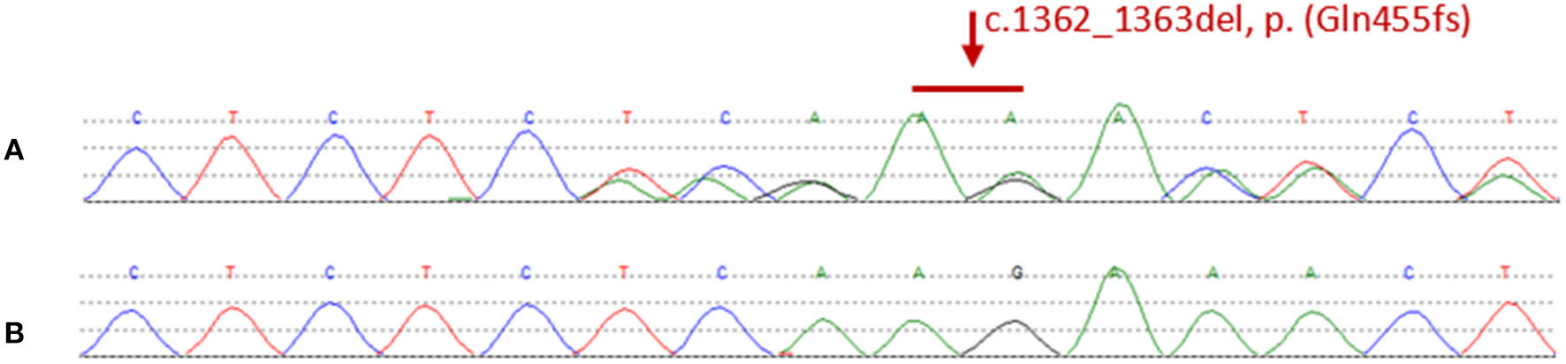
**(A)** Representative sequencing results from a patient in the family. The graph shows a “c.1362_1363del” deletion mutation of exon 14 in krit1. **(B)** Representative sequencing results from a normal member without mutation in the family.

We found a heterozygous deletion mutation (frameshift mutation) in the *KRIT1/CCM1* gene of the proband, which we descrbied as c.1362_1363del (p.Gln455fs), in contrast, sequence analysis of *CCM2* and *CCM3* did not reveal any pathogenic mutations. At the same time, we also analyzed the *KRIT1/CCM1* sequence of his father, son, sister and niece, and found the same mutation in all family members whose MRI scan showed cavernous hemangioma. The c.1362_ 1363del is a type of frame-shift mutation found in the patient's DNA (Figure 3), which alters the reading frame of the *KRIT1/CCM1* gene, leading to a premature stop codon.

## Discussion

Cavernous hemangioma is a type of hemangioma that usually occurs in young adults and rarely occurs in older adults (13). The incidence of this condition in men and women appears to be equal, although, in some reports, women with cavernous hemangioma exhibit a higher bleeding rate (14). In the current family, five patients had bleeding, including two men and three women, which is different from the findings of other studies. There are two known manifestations of intracranial cavernous hemangioma: one is sporadic, usually with isolated lesions, and the other is a familial disease, which usually has multiple lesions and is inherited in an autosomal dominant nature with incomplete penetrance. In most cases, CCMs are located inside the brain, and 40% of patients have no obvious symptoms, while about 60% will experience generalized or focal seizures, focal neurological deficits, headaches and other symptoms of intracranial hemorrhage. The risk of symptomatic bleeding varies depending upon the location of the cavernoma, and is generally greater for lesions deeper in the brain (4). The risk of bleeding recurrence in each patient every year increases from 5 to 60% (15). Therefore, it is necessary to treat lesions early on after the first seizure or bleeding event. Among the cases of intracranial cavernous hemangioma caused by *CCM1, CCM2*, and *CCM3* gene mutations, only reports of *CCM1* mutations accompanied by cutaneous capillary—venous malformations have been published (16), and there is no cavernous hemangioma patient with skin venous—capillary malformations in the family detailed in this report. According to reports, the coexistence of brain CCM and spine CCM is very rare (17), and there was no such patient in the family involved in this report.

MRI is the most sensitive method for detecting intracranial CCM lesions. According to the results of MRI examination, CCM can be divided into four types (18). Type 1 lesions show high-intensity signals on both T1 and T2 sequences. Type 2 lesions are characterized by a mixed signal of high and low signals surrounded by low signal edges and are the most common CCM lesions. Type 3 lesions show low signals on both T1 and T2 sequences. Finally, type 4 lesions are low-signal lesions that can only be detected in radio-echo sequences. One of the main characteristics of both symptomatic and asymptomatic mutation carriers is whether there are multiple lesions on the brain MRI scan (7). Among the family members involved in this report, the brain MRI of mutation carriers also showed multiple lesions. When there are multiple lesions, gradient-echo sequences are more sensitive than turbo spin-echo (TSE) sequences; however, on TSE and gradient echo MRI sequences, some mutation carriers may not show any lesions (19). Therefore, it is necessary to use systematic gradient-echo sequences to detect patients suspected of having CCM. In patients with hemorrhagic metastases, the diagnosis may become difficult; moreover, if the peripheral edema causes a contrast enhancement effect after bleeding, rapid recurring bleeding occurs, or the lesions increase, the diagnosis will be more difficult. If there is doubt about a diagnosis, surgical removal of the lesions can be considered. For an uncertain or doubtful diagnosis, a biopsy can be performed by surgically removing the lesion. However, due to the risk of bleeding, a biopsy is usually not recommended.

Our findings demonstrate that not only incomplete clinical but also radiological penetrance precludes the use of cerebral MRI to firmly establish a non-carrier status even in adults and using highly sensitive gradient-echo sequences. In this case series, we found that we cannot rely solely on cranial MRI to determine whether a person is a non-mutant carrier, even in adults or using highly sensitive gradient echo sequences because of the clinical and radiological incomplete appearance. According to prior reports, based on the results of MRI examinations, about 75% of patients with significantly isolated CCM will have a parent with neuroradiological changes (8). Therefore, we infer that only those family members who carry familial cavernous hemangioma mutant genes require surveillance and medical management and intervention for symptoms and lesions.

In the past decade, the incidence of familial cavernomas hemangioma in Hispanic American (20) and Chinese families (21) has been higher than that in other populations, and existing clinical reports are mainly concentrated on these two groups of people.

In this study, we found a case of FCCM through a proband from a Chinese family. Patients from this family showed a series of clinical symptoms and features, including seizures, language disorders, dyskinesia, and other symptoms resulting from multiple brain lesions and bleeding. In this case, a heterozygous frame-shift mutation, which has been reported by several times in other countries in the past (22–25), was found in exon 14 (c.1362_1363del) of *CCM1*. This single-stranded deletion was predicted to cause coding disorder of amino acid Gln at position 455, leading to premature appearance of stop codons, making the encoded protein truncated and losing normal function.

The *CCM1* gene with 16 coding exons encodes the Krit1 protein (containing 736 amino acids, which has three ankyrin domains and a FERM domain). Mutations in *CCM1* all generate premature stop codons (26). So far, more than 90 different *CCM1* mutations have been reported (27). It is speculated that these mutations in the CCM1 gene can lead to abnormal mRNA, which in turn leads to non-functional proteins (28). Familial CCM mutations are inherited in an autosomal dominant manner, of which the penetrance is incomplete at both the clinical and neuroradiological levels (29). Therefore, clinically asymptomatic “carrier” individuals with a disease-causing mutation should be monitored for lesions by MRI (30). In Japan, a recent survey showed genomic differences between different ethnic groups (31), where the ratio of *CCM1, CCM2*, and *CCM3* mutation rates may reflect the unique genomic characteristics of the Japanese population (32).

The form of genetic mutation discovered in this study is a type of *CCM1* mutation which traditionally leads to the premature generation of stop codons, resulting in truncated proteins. Several reports on FCCM caused by *CCM1* mutations in China have published in recent years, as shown in Table 3. One point mutation (c.2835CT, p.Q698X) in exon 19 caused the stop codon (Q698X) to be generated prematurely, which resulted in a truncated Krit1 protein (11). Another point mutation (c.1298CG, p.S430X) in exon 14 caused the stop codon to be generated prematurely, which produced another truncated form of the Krit1 protein (12). A deletion mutation of the AT frameshift at NTs 1292 and 1293 in exon 13 destroyed the Krit1 protein-encoding mechanism (21). The splicing mutation of the GTA deletion of the intron 9/exon 10 receptor splice site of the *CCM1* gene produced a premature stop codon at the 23rd amino acid, which led to the truncation of the Krit1 protein (33). A deletion mutation (c.1197delCAAA) in exon 12 led to an early stop codon (TGA) at NT 1,228, which produced a truncated Krit1 protein of only 409 amino acids (28). One T—deletion mutation in exon 14 (c.1396delT) caused a premature stop codon in the second half of the *CCM1* gene, resulting in a Krit1 protein of only 493 amino acids in length (34). A heterozygous T deletion in exon 15 (c.1542delT) of the 16 coding exons (exons 5–20) led to an early stop codon, producing a truncated Krit1 protein of 526 amino acids (35). Detection of DNA using exome - capture sequencing technology revealed a c.1159G>T transition in exon 14 of the *CCM1* gene, which led to premature termination at codon 387 (p. E387*) (36). A T—insertion mutation in exon 18 (c.1896_1897insT) caused a frameshift at NTs 1896 and 1897, which destroyed the coding of Krit1 protein (37). A novel heterozygous nonsense NT transition (c.1864C>T; p.Gln622X) in exon 17 of the *CCM1/KRIT1* gene was predicted to trigger a premature stop codon (TAG) at NTs 1864 to 1866 to generate a truncated Krit1 of 621 amino acids in length (30). DNA sequencing analysis of the proband disclosed a novel heterozygous deletion mutation (c.1919delT; p.Phe640SerfsX21) in exon 17 of the *CCM1* gene. This mutation led to a frameshift and caused a premature termination codon to generate a truncated Krit1 of 659 amino acids (38). Finally, a deletion frameshift mutation, c.1635delA (p.Thr545fsTer6), in the *CCM1* gene, produced a truncated protein lacking 191 (546–736) amino acids at the C-terminal of the Krit1 protein (27).

**Table 3:** Reports of 12 Chinese articles on FCCM in the past 10 years.

**Year**	**Site**	**Form of Mutation**	**The length of the truncated protein**
2002	exon 19	point mutation (c.2835CT, p.Q698X)	698 amino acids
2003	exon 14	point mutation (c.1298CG, p.S430X)	429 amino acids
2005	exon 13	deletion mutation of the A-T frameshift at NT 1292 and 1293	433 amino acids
2006	exon 10/intron 9	splicing mutation of the GTA deletion	418 amino acids
2011	exon 12	deletion mutation (c.1197delCAAA)	409 amino acids
2013	exon 14	T deletion mutation(c.1396delT)	493 amino acids
2014	exon 15	heterozygous T deletion in exon 15 (c.1542delT)	526 amino acids
2015	exon 14	c.1159G>T transition (p.E387+)	387 amino acids
2017	exon 18	T insertion mutation in exon 18 (c.1896_1897insT)	633 amino acids
2017	exon 17	heterozygous nonsense nucleotide transition	621 amino acids
2018	exon 17	(c.1864C>T;p.Gln622X)	659 amino acids
2020	exon 15	heterozygous deletion mutation (c.1919delT; p.Phe640SerfsX21)	545 amino acids

In summary, we report a case series of FCCM in a Chinese family with a frame-shift mutation of *CCM1* that has been previously reported at abroad, and we report the corresponding classical genetic characteristics, clinical characteristics and imaging characteristics. Our findings further deepen the understanding of *CCM* mutations and the associated clinical phenotypes. Moreover, our findings is beneficial knowledge to support genetic counseling of FCCM patients.

## Conclusions

The study demonstrates Chinese FCCM with the *KRIT1/CCM1* insertion mutation, here in the course of this trial, we have collected three generations of families with this mutation, and then we perfected the clinical symptoms and signs caused by this mutation of the gene, as well as the corresponding imaging changes. The results of this trial investigation are very consistent with the classic FCCM in terms of clinical manifestations, imaging manifestations, or genetic manifestations, which deepen the understanding of CCM mutations and the associated clinical phenotypes. Compared with previous reports, the investigation results of this clinical experiment further show that the gene mutation form we reported plays an important role in human familial cavernous hemangioma. Moreover, it will be of significance in genetic counseling for CCM.

## Data Availability Statement

The datasets presented in this article are not readily available due to ethical and privacy restrictions. Requests to access the datasets should be directed to the corresponding author.

## Ethics Statement

The studies involving human participants were reviewed and approved by the Ethical Committee of Qilu Hospital. Written informed consent to participate in this study was provided by the participants' legal guardian/next of kin.

## Author Contributions

XW and BH: study design. WL and ML: data collection. WL, ML, JW, DL, ZC, XL, and ZF: statistical analysis. WL, ML, JW, DL, and ZC: data interpretation. WL, ML, JW, DL, ZC, and XL: manuscript preparation. XW, BH, and JW: literature search. XW: funds collection. All authors contributed to the article and approved the submitted version.

## Conflict of Interest

The authors declare that the research was conducted in the absence of any commercial or financial relationships that could be construed as a potential conflict of interest.

## Publisher's Note

All claims expressed in this article are solely those of the authors and do not necessarily represent those of their affiliated organizations, or those of the publisher, the editors and the reviewers. Any product that may be evaluated in this article, or claim that may be made by its manufacturer, is not guaranteed or endorsed by the publisher.
